# 

**Published:** 1907-10-12

**Authors:** 


### CONTENTS.

| | PAGE |
|---|---|
| **The Grip of the Specialist** | 27 |
| **The Prevalence of Drug Habits** | 28 |
| **Annotations—** | |
| Malta Fever—The Medical Societies—The Midwives Act | 29 |
| **Medical Opinion and Movement** | 30 |
| **Hospital Clinics—** | |
| How to Perform a Post-mortem Examination: The Experience of a Lifetime, and its Practical Teaching. By Sir Henry D. Littlejohn, M.D., LL.D. (Edin.) | 31 |
| **Special Article—** | |
| What is a Disease: What is the Meaning of Diagnosis? | 34 |
| **Illustrative Cases—** | |
| Cervico-Facial Actinomycosis | 37 |
| Raynaud's Disease Affecting Tongue as well as Extremities | 38 |
| **Points in Surgery—** | |
| The Treatment of Fractures from a Common-sense Point of View | 39 |
| The Operative Treatment of Internal Piles | 40 |
| **Dermatology—** | |
| Pemphigus | 41 |
| **Gynæcology—** | |
| Some Remarks on Puerperal Sapræmia | 42 |
| **Laryngology and Rhinology—** | |
| The Treatment of Frontal Sinusitis | 43 |
| **Points in Pathology—** | |
| Pathology for General Practitioners | 44 |
| **Public Health and Hygiene—** | |
| Diagram of the Weekly Death Rate in 1907 | 45 |
| **New Appliances and Things Medical** | 46 |
| **Hospital Administration—** | |
| Where is "That Man from Sheffield"? | 47 |
| The Bristol Royal Infirmary Impasse | 48 |
| Social and Poor-law Problems | 49 |
| News and Coming Events | 50 |
| **Nursing Administration—** | |
| The Nurses' Home | 51 |
| The Common Task | 52 |

